# Effects of Partial Replacement of Wheat Bran with Poplar Wood Composite Fiber on Growth Performance, Nutrient Apparent Digestibility, Immune Function, and Gut Microbiota in Growing Pigs

**DOI:** 10.3390/vetsci13060588

**Published:** 2026-06-17

**Authors:** Yuyang Fan, Ge Gao, Xinyue Jiang, Dongxu Ming, Yanpin Li, Wenjuan Sun, Xilong Li, Yu Pi

**Affiliations:** Key Laboratory of Feed Biotechnology of the Ministry of Agriculture and Rural Affairs, Institute of Feed Research, Chinese Academy of Agricultural Sciences, Beijing 100081, China; w18832417225@163.com (Y.F.); 82101192355@caas.cn (G.G.); j15613565090@163.com (X.J.); mingdongxu@caas.cn (D.M.); liyanpin@caas.cn (Y.L.); sunwenjuan@caas.cn (W.S.)

**Keywords:** dietary fiber, poplar wood composite fiber, wheat bran replacement, growth performance, nitrogen metabolism, gut microbiota, growing pigs

## Abstract

The objective of this study was to evaluate the effects of partially replacing wheat bran with poplar wood composite fiber (PWCF) on growth performance, immune status, apparent total tract digestibility (ATTD), and gut microbial composition in growing pigs. A total of 140 healthy crossbred (Duroc × Landrace × Yorkshire) growing pigs with an initial body weight of 47.25 ± 0.49 kg were randomly assigned to two dietary treatments, with five replicates per treatment and fourteen pigs per replicate. The control (CT) group was fed a corn–soybean meal-based diet containing wheat bran and rice bran meal, whereas the experimental group received the same diet in which 2% wheat bran was replaced by PWCF. The experiment lasted for 60 days. Compared with the CT group, replacing wheat bran with PWCF did not affect body weight, average daily feed intake, feed conversion ratio, or average daily gain on days 30 or 60 (*p* > 0.05). In addition, no negative effects were observed on ATTD of nutrients and serum immunoglobulin A (IgA), IgG, and IgM levels at either time point, indicating that PWCF can serve as a suitable partial substitute for wheat bran in growing pig diets. However, it could regulate nitrogen metabolism by reducing blood urea nitrogen (BUN) concentration and the BUN/creatinine ratio, as well as decreasing total free amino acids in serum (*p* < 0.05). In addition, the antioxidant capacity can be transiently improved by increasing catalase activity. Gut microbiota analysis showed that the replacement significantly increased the relative abundances of *Treponema*, the Lachnospiraceae_XPB1014_group and Prevotellaceae_UCG-001 (*p* < 0.05). These changes suggest that PWCF modulates gut microbiota and enriches fiber-degrading bacterial populations. Overall, substituting wheat bran with PWCF did not impair growth performance, immunity, or digestibility, while altering microbial community composition. These findings support the potential application of PWCF as an alternative fiber source, contributing to greater diversity in feed formulation.

## Simple Summary

This study investigated the effects of replacing wheat bran with poplar wood composite fiber (PWCF) in pig diets. The research involved 140 growing pigs fed either a control diet or an experimental diet with 2% wheat bran replaced by PWCF for 60 days. The results showed that PWCF did not negatively affect pig growth performance, nutrient digestibility, or immune function. Instead, it helped regulate nitrogen metabolism by lowering blood urea nitrogen (BUN) levels and serum total free amino acids (TFAAs) and enhanced antioxidant capacity by increasing catalase activity. Notably, PWCF supplementation led to significant increases in the relative abundance of certain gut bacteria known to degrade dietary fiber. Overall, PWCF can be a suitable alternative fiber source in pig diets, offering benefits such as nitrogen metabolism regulation and gut microbiota modulation, without compromising growth or health. This supports the use of PWCF in diversifying fiber ingredients in pig feed.

## 1. Introduction

Dietary fiber (DF) refers to a group of food components that are resistant to hydrolysis by endogenous digestive enzymes and are therefore poorly digested and absorbed in the small intestine. It is widely present in plant-derived foods such as cereals, fruits, and vegetables [1]. In recent years, increasing evidence has shown that DF can influence growth performance [2], immune function [3], apparent total tract digestibility (ATTD) [4], and gut microbial composition in growing pigs [5]. Previous studies have demonstrated that moderate inclusion of DF in diets does not impair average daily gain or overall growth performance in growing pigs [6,7]. However, supplementation with a high level of soybean hulls (20%) has been reported to reduce the gain-to-feed ratio [8]. Previous studies have also shown that DF supplementation can improve ileal nutrient digestibility [7] while reducing the ATTD of crude protein (CP) and energy [9]. In addition, DF has been reported to promote intestinal development and support gut health [10,11]. Similar findings indicate that DF inclusion may decrease meal frequency while increasing average meal size and reducing the apparent fecal digestibility of dry matter (DM), organic matter, crude ash, nitrogen, and gross energy (GE). However, it may enhance the digestibility of crude fiber (CF) and neutral detergent fiber (NDF) [12]. Collectively, these results suggest that the effects of DF in pig diets are complex and may depend on the inclusion level and fiber source. Therefore, further studies are required to determine the optimal DF supplementation level for growing pigs.

Wheat bran is a widely used feed ingredient due to its broad availability and low cost. It is rich in dietary fiber (approximately 35–45%), crude protein (14–18%), and minerals (ash, 4–8%). The dietary fiber fraction is predominantly insoluble, with insoluble dietary fiber accounting for approximately 90–95% of total dietary fiber and soluble dietary fiber representing only 5–10% [13,14]. However, the application of wheat bran in animal diets also has notable limitations. During storage, wheat bran is susceptible to contamination by pathogenic microorganisms and the production of various mycotoxins, such as deoxynivalenol and zearalenone. These toxins can impair immune function and exert chronic negative effects on growth performance, as well as reproductive health [15]. Wheat bran has been reported to harbor relatively high microbial loads. The microorganisms commonly detected in wheat bran include bacterial genera such as *Pseudomonas aeruginosa*, *Micrococcus*, and *Lactobacillus*, as well as fungi including *Aspergillus*, *Penicillium*, and *Streptomyces*, together with their associated mycotoxins. This microbial contamination increases the difficulty of wheat bran storage and poses potential risks to feed safety [16]. As an alternative lignocellulosic fiber source, poplar wood composite fiber (PWCF) is produced from poplar wood through mechanical processing and fiber separation [17]. It is primarily composed of structural carbohydrates, including cellulose, hemicellulose, and lignin, and is characterized by a high dietary fiber content as well as favorable physicochemical properties, such as water-holding and swelling capacities. Owing to the extensive cultivation of poplar trees and the large quantity of wood-processing residues generated annually, PWCF represents an abundant and potentially sustainable fiber resource. Furthermore, PWCF is predominantly composed of insoluble dietary fiber, with only a small proportion of soluble dietary fiber, making it a valuable source of structural fiber in animal diets. These characteristics suggest that PWCF may serve as a promising alternative fiber source to partially replace conventional fiber ingredients such as wheat bran in swine diets. However, information regarding its application and physiological effects in pigs remains limited.

Therefore, in the present study, a poplar wood-based composite fiber was developed using poplar powder as the main ingredient, and its feasibility as a partial substitute for wheat bran was evaluated. The present study aimed to evaluate the effects of dietary supplementation with PWCF on growth performance, immune status, ATTD, and gut microbial composition in growing pigs. By examining these parameters, this study sought to determine whether PWCF could partially replace wheat bran in growing pig diets. Such substitution may contribute to reducing feed costs and improving feed efficiency, thereby providing a theoretical basis for the practical application of alternative DF sources in swine nutrition.

## 2. Materials and Methods

The experiment was conducted at the Tianpeng Experimental Farm located in Langfang, China, from July 2022. All animal procedures in this study were approved by the Animal Care and Use Committee of the Feed Research Institute, Chinese Academy of Agricultural Sciences (IFR-CAAS20220725).

### 2.1. Experimental Design and Animal Management

A total of 140 healthy growing pigs (Duroc × Landrace × Yorkshire), with an equal number of barrows and gilts and a similar initial body weight (47.25 ± 0.49 kg), were randomly assigned to two dietary treatment groups. Each treatment included five replicates, with fourteen pigs per replicate. The control group (CT) was fed the basal diet, whereas the fiber treatment group (FF) received the same diet in which 2% wheat bran was replaced with 2% PWCF (Hebei Weierli Animal Pharmaceutical Group Co., Ltd., Shijiazhuang, China). The basal diet was formulated as a pelleted feed according to the nutrient requirements for growing–finishing pigs recommended by the NRC (2012), and its nutrient composition is presented in Table 1. The PWCF is composed of 50% fiber sourced from white poplar harvested between May and July 2022. The white poplar was ground to achieve a fiber particle size ranging from 300 to 400 μm. This material was thoroughly mixed with the remaining 50% of the composition, which primarily consists of maifanstone, xanthan gum, and montmorillonite as carrier components. The hygienic quality of the PWCF complies with the Chinese National Feed Standard GB 13078-2017 [18]. The nutrient composition of the PWCF used is shown in Table 2. The experimental period lasted for 60 days.

### 2.2. Growth Performance

Body weight (BW) was recorded individually for each pig on days 0, 30, and 60 using a calibrated electronic scale (accuracy: ±0.1 kg). Feed intake was measured simultaneously. Average daily gain (ADG), average daily feed intake (ADFI), and the feed-to-gain ratio (F: G) were calculated to evaluate growth performance.

### 2.3. Determination of Serum Biochemical, Immunoglobulins, and Antioxidant-Related Indices

At the end of the experiment, one pig from each replicate was selected for blood sampling; blood samples were collected from the anterior vena cava of the experimental pigs using anticoagulant blood collection tubes, and the samples were centrifuged at 3000× *g* and 4 °C for 15 min; the supernatant was collected for the determination of serum biochemical, immunoglobulins, and antioxidant-related indices. Serum levels of glucose (GLU), triglyceride (TG), total protein (TP), and blood urea nitrogen (BUN) were measured using commercial assay kits from Chengdu Makalu Biotechnology Co., Ltd., Chengdu, China, on an automatic biochemical analyzer (Erba XL-200; Erba Diagnostics, Mannheim, Germany), with the specific procedures carried out following the corresponding kit instructions.

Blood ammonia and creatinine were determined using a commercial assay kit (Beijing Boxbio Science & Technology Co., Ltd., Beijing, China). Serum amino acids were analyzed using a high-performance liquid chromatography system (LC-20AT; Shimadzu Corporation, Kyoto, Japan). Immunoglobulins in serum, including immunoglobulin A (IgA), immunoglobulin G (IgG), and immunoglobulin M (IgM), were detected with assay kits purchased from Shanghai Enzyme-Linked Biotechnology Co., Ltd. (Shanghai, China), and the specific operations were performed in accordance with the kit instructions. Serum catalase (CAT), superoxide dismutase (SOD), and malondialdehyde (MDA) were measured using commercial assay kits (Nanjing Jiancheng Bioengineering Institute, Nanjing, China) according to the manufacturers’ instructions.

### 2.4. Nutrient Composition and Fiber Physical Characteristics Determination, and Apparent Total Tract Digestibility Calculation

During the last three days of the experiment, fresh fecal samples were collected by rectal stimulation from three randomly selected pigs in each pen. Approximately 200 g of feces was obtained from each pig, and samples collected from pigs within the same pen were thoroughly mixed to generate one composite sample per replicate. The composite fecal samples were dried in a laboratory oven at 65 °C for 48 h to obtain air-dried material for subsequent analyses. Dry matter (DM), CP, EE, Ca, and ash content in both feed and fecal samples were analyzed using AOAC official methods 930.15, 990.03, 920.39, 967.30, and 942.05, respectively. Gross energy (GE) was determined via adiabatic bomb calorimetry (Parr 6300, Parr Instrument Company, Moline, IL, USA). The contents of NDF and acid detergent fiber (ADF) were quantified according to the protocols described by Van Soest [19]. Furthermore, the water-holding capacity and swelling capacity of poplar wood composite fiber were determined according to the methods described by previous studies [20,21]. Acid-insoluble ash (AIA) was used as an indigestible marker, and the apparent digestibility of dietary nutrients was evaluated according to the procedures of the Association of Official Analytical Chemists (AOAC, 942.05) [22].

The ATTD was calculated using the following formula:

ATTD = [1 − (A_feed_ × N_feces_)/(N_feed_ × A_feces_)] × 100,

where A_feed_ = Content of AIA in feed (%); A_feces_ = Content of AIA in feces (%); N_feed_ = Content of a certain nutrient in feed (%); and N_feces_ = Content of a certain nutrient in feces (%).

### 2.5. Fecal Microbiota Analysis

On the final day of the experiment, fresh fecal samples were collected via rectal massage and then frozen and stored at −80 °C for subsequent analysis. Following the procedures described in a previous study [23], total microbial DNA was extracted from fecal samples using a commercial kit (Omega Bio-Tek, Norcoss, GA, USA) according to the manufacturer’s instructions. The V3–V4 hypervariable region of the bacterial 16S rRNA gene was amplified using the universal primers 338F (5′-ACTCCTACGGGAGGCAGCAG-3′) and 806R (5′-GGACTACHVGGGTWTCTAAT-3′). The PCR products were purified with a DNA purification kit (Axygen Biosciences, Union City, CA, USA). Purified amplicons were sequenced on a high-throughput sequencing platform. Sequence data were processed using the DADA2 pipeline to denoise reads and generate amplicon sequence variants (ASVs) under default parameters. Taxonomic assignment of all ASVs was performed in QIIME 2 using a naïve Bayes classifier against the SILVA 138 reference database. Alpha diversity indices were calculated using Mothur (version 1.30), whereas beta diversity was analyzed using the vegan package (version 3.3.1). Principal coordinate analysis (PCoA) based on Bray–Curtis distances was conducted to visualize differences in microbial community composition, and analysis of similarities (ANOSIM) was applied to assess the statistical significance of group separation. Taxonomic differentiation was identified through LEfSe (linear discriminant analysis effect size). All bioinformatic analyses were performed using the Majorbio Cloud Platform (https://cloud.majorbio.com/) (accessed on 19 February 2026) (Majorbio Bio-Pharm Technology Co., Ltd., Shanghai, China).

### 2.6. Statistical Analysis

All experimental data were analyzed using SAS statistical software (version 9.4; SAS Institute Inc., Cary, NC, USA). The experiment was conducted using a completely randomized design with two dietary treatments and five replicate pens per treatment. For growth performance variables (BW, ADG, ADFI, and F: G), the pen was considered the experimental unit (*n* = 5 pens per treatment). For apparent total tract digestibility (ATTD), fecal samples collected from pigs within the same pen were pooled to generate one composite sample per pen; therefore, the pen was considered the experimental unit (*n* = 5 pens per treatment). For serum biochemical parameters, amino acid profiles, immunoglobulins, antioxidant indices, and fecal microbiota analyses, one pig was randomly selected from each pen for sampling, resulting in five observations per treatment group (*n* = 5 pens per treatment). Body weight was measured on days 30 and 60 and analyzed separately at each sampling time to evaluate treatment effects during the corresponding production phase. Therefore, repeated-measures analysis was not performed. Data were inspected prior to analysis to verify that they were suitable for parametric testing. Differences between treatments were evaluated using Student’s *t*-test. No random effects were included in the statistical model because treatment was the only factor of interest and the pen was considered the experimental unit. Results are presented as mean ± SEM, and statistical significance was declared at *p* < 0.05. Given the limited number of replicate pens per treatment (*n* = 5), the statistical power to detect small treatment effects may be limited.

## 3. Results

### 3.1. Growth Performance and Apparent Total Tract Nutrient Digestibility

Dietary supplementation with PWCF had no significant effects on BW or F: G in growing pigs (*p* > 0.05) (Table 3). In addition, during the period from days 31 to 60, pigs in the FF group exhibited slightly higher ADG and ADFI compared with the CT group, although these differences were not statistically significant (*p* > 0.05). As shown in Table 4, no significant differences were observed between the FF group and CT group in the apparent digestibility of DM, CP, calcium, P, EE, or GE (*p* > 0.05).

### 3.2. Serum Biochemical Parameters

There were no significant differences observed in serum GLU, TC, TP, creatinine, or blood ammonia concentrations between the FF group and the CT group on either day 30 or day 60 (*p* > 0.05) (Table 5). However, on day 60, serum BUN levels tended to be lower in the FF group than in the CT group (*p* = 0.084). In addition, the BUN/creatinine ratio was not significantly affected on day 30 (*p* > 0.05), whereas it was significantly lower in the FF group than in the control group on day 60 (*p* < 0.05).

### 3.3. Serum-Free Amino Acid Profiles

As shown in Table 6, on day 30, no significant differences were observed in the serum concentrations of aspartic acid, glutamic acid, serine, histidine, glycine, threonine, arginine, alanine, tyrosine, valine, methionine, isoleucine, lysine, or leucine between the FF group and the CT group (*p* > 0.05). However, the concentrations of phenylalanine and total free amino acids (TFAA) tended to be lower in the FF group than in the CT group (*p* = 0.082 and *p* = 0.059, respectively). On day 60, no significant differences were observed in the concentrations of aspartic acid, glutamic acid, serine, histidine, glycine, arginine, tyrosine, valine, methionine, isoleucine, phenylalanine, lysine, or leucine between the two groups (*p* > 0.05). However, the FF group showed significantly lower concentrations of threonine and TFAA than the CT group (*p* < 0.05), and alanine tended to be decreased (*p* = 0.065).

### 3.4. Serum Immunoglobulin Levels and Antioxidant-Related Indices

As shown in Table 7, no significant differences were observed in the serum concentrations of IgG, IgA, or IgM between the FF group and CT group, both on day 30 and day 60 (*p* > 0.05). As for the antioxidant-related indices, as shown in Table 8, on day 30, compared with the control group, the FF group significantly increased the serum CAT concentration in growing pigs (*p* < 0.05), but had no significant effects on MDA or SOD levels (*p* > 0.05). However, on day 60, no significant differences were observed in serum MDA, CAT, or SOD levels between the FF group and the control group.

### 3.5. Fecal Microbiota Composition

**Figure 1:**
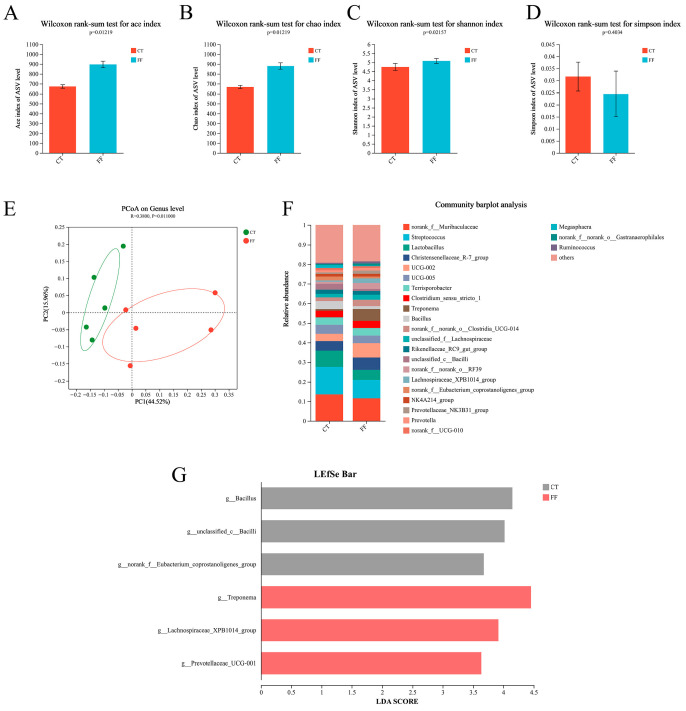
Effects of partial replacement of wheat bran with poplar wood composite fiber on fecal microbial composition in growing pigs. (**A**) ACE index; (**B**) Chao1 index; (**C**) Shannon index; (**D**) Simpson index; (**E**) PCoA based on Bray–Curtis distances at the genus level; (**F**) Microbial community composition at the genus level; (**G**) LEfSe analysis of differential enrichment of fecal bacteria at the genus level (linear discriminant analysis [LDA] > 3.5). CT = basal diet; FF = CON + 2% poplar wood composite fiber (replacing 2% wheat bran). *n* = 5.

Figure 1 illustrates the effects of partial replacement of wheat bran with PWCF on the fecal microbiota of growing pigs. After 60 days of feeding, the ACE and Chao1 indices were significantly higher in the FF group than in the CT group (*p* < 0.05; Figure 1A, B). Similarly, the Shannon diversity index was slightly increased in the FF group compared with the CT group (*p* < 0.05; Figure 1C). In contrast, the Simpson index was numerically higher in the CT group than in the FF group, although the difference was not statistically significant (*p* > 0.05; Figure 1D). Principal coordinate analysis (PCoA) based on Bray–Curtis distances further revealed a significant separation of microbial communities between the FF and CT groups at the genus level (ANOSIM: *p* = 0.011, R = 0.380; Figure 1E), indicating that PWCF supplementation markedly altered the overall gut microbial structure.

In addition, the genus-level taxonomic composition of the fecal microbiota was analyzed. The top 10 most abundant genera across treatments included norank_f_Muribaculaceae, *Streptococcus*, *Lactobacillus*, Christensenellaceae_R-7_group, UCG-002, UCG-005, *Terrisporobacter*, *Clostridium*_*sensu*_stricto_1, *Treponema*, and *Bacillus* (Figure 1F). LEfSe analysis revealed distinct microbial biomarkers between the CT and FF groups (Figure 1G). Taxa enriched in the FF group mainly included *Treponema*, the Lachnospiraceae_XPB1014_group and Prevotellaceae_UCG-001. In contrast, *Bacillus*, unclassified Bacilli, and the norank_f_*Eubacterium coprostanoligenes* group were identified as biomarkers of the CT group.

## 4. Discussion

In traditional nutrition research, DF has long been regarded as an anti-nutritional factor because it cannot be degraded by endogenous digestive enzymes and may reduce nutrient digestibility. Therefore, diets formulated for monogastric animals, particularly growing pigs, have typically contained relatively low levels of DF, generally ranging from approximately 8% to 15% total dietary fiber depending on the production stage and feeding objective [24,25]. Although monogastric animals cannot directly utilize DF, gut microorganisms possess the capacity to ferment fiber substrates, producing beneficial microbial metabolites that may exert probiotic effects on the host [25]. At present, the application of DF in swine nutrition remains challenging, as multiple factors must be considered, including the wide variety of fiber sources, the complexity of fiber composition, and differences among pig breeds [26]. In this study, wheat bran was partially replaced with PWCF to assess the effects of dietary fiber supplementation on growth performance, immune status, nutrient digestibility, and gut microbiota in growing pigs.

### 4.1. Partial Replacement of Wheat Bran with PWCF Did Not Affect Growth Performance in Growing Pigs

DF levels in pig diets are often restricted because of their potential anti-nutritional properties, which may reduce the digestibility of protein and energy [27]. In the present study, partial replacement of wheat bran with PWCF did not adversely affect average daily gain or feed conversion ratio in growing pigs. These findings suggest that PWCF can be incorporated at a low inclusion level without compromising growth performance. Consistent with our results, Wang et al. reported that pigs fed diets containing 5%, 10%, or 15% alfalfa meal exhibited improved feed utilization efficiency, whereas ADFI and ADG were not significantly affected [28]. Together, these results indicate that moderate inclusion of predominantly insoluble lignocellulosic fiber sources, such as PWCF, can be well tolerated by growing pigs and does not necessarily impair productive performance under practical feeding conditions. Consistent with the present findings, previous studies have reported that moderate inclusion of insoluble dietary fiber sources, including wheat bran, sugar beet pulp, and soybean hulls, generally does not impair growth performance in growing pigs when total dietary fiber levels are maintained within practical feeding ranges. The increased utilization of threonine for mucin synthesis may be particularly relevant in the present study, as PWCF supplementation improved intestinal health indicators, suggesting enhanced mucus barrier activity and epithelial maintenance. However, other research has reported that high-fiber diets may reduce ADG and increase the F: G. For instance, when DF levels increased from 5% to 7%, ADG was significantly decreased in growing pigs [29,30]. These results indicate that excessive DF inclusion can exert negative effects on growth performance. Similarly, pigs fed high-fiber diets have been reported to exhibit poorer growth outcomes, including reduced carcass weight and dressing percentage, as well as a higher carcass fat iodine value [31]. Such adverse effects may be attributed to the fact that DF can increase digesta viscosity and limit interactions between nutrients and digestive enzymes in the small intestine, thereby reducing nutrient digestion and absorption [32].

### 4.2. Partial Replacement of Wheat Bran with PWCF Did Not Affect the Apparent Total Tract Digestibility of Nutrients in Growing Pigs

Apparent total tract digestibility is an important indicator for evaluating feed efficiency, improving swine production performance, and reducing environmental burden [33]. Previous studies have shown that increasing DF levels to 6.86% significantly decreased the digestibility of CP, EE, CF, and ADF in growing pigs [34]. Similarly, in corn-based diets, increasing DF content resulted in a linear reduction in the ATTD of CP, DM, ash, and organic matter [35]. This decline may be attributed to the increased proportion of plant cell wall components associated with DF sources, which are generally resistant to digestion [36]. Other studies have also reported that the digestibility of DM, GE, and NDF decreases as DF levels increase in growing pig diets [37]. In addition, DF fractions have been negatively correlated with DE and metabolizable energy concentrations, indicating that high-fiber diets may reduce energy utilization in pigs [38]. However, in the present study, supplementation with PWCF did not exert any negative effects on ATTD, suggesting that this fiber source can effectively substitute wheat bran at the tested inclusion level. It is noteworthy that PWCF contained a relatively high ash content (30.13%) compared with many conventional dietary fiber sources. This characteristic may reflect the naturally occurring mineral fraction associated with poplar wood and the compositional properties of the processed fiber product. Despite the elevated ash content, no adverse effects were observed on nutrient digestibility, growth performance, serum biochemical parameters, or intestinal health indicators in the present study. These findings suggest that the mineral fraction of PWCF was well tolerated at the dietary inclusion level evaluated. Nevertheless, further characterization of the mineral composition and bioavailability of PWCF is warranted to better understand its nutritional contribution and potential physiological implications.

### 4.3. Effects of Partial Replacement of Wheat Bran with PWCF on Immune Function and Serum Biochemical Parameters in Growing Pigs

The content of immunoglobulins in serum can directly reflect the immune capacity of the organism. In the present study, supplementation of PWCF in the diet to partially replace wheat bran exerted no adverse effects on the immune capacity of growing pigs. However, several studies have shown that high-fiber diets reduce the contents of IgM and IgG in the plasma of growing pigs, impair their immune capacity, and induce certain intestinal damage [26]. Relevant studies have demonstrated that TP is closely associated with protein absorption and utilization [39]. Meanwhile, GLU, TG, LDL, HDL, TC, and other lipid metabolism indices are closely associated with fat deposition and metabolism in pigs [40]. Previous studies have shown that reductions in serum glucose and triglyceride concentrations are often observed in diets containing high levels of soluble dietary fiber, which can increase digesta viscosity and slow the diffusion and absorption of glucose and lipids across the intestinal epithelium [41,42]. In the present experiment, supplementation of PWCF in the diet did not reduce the contents of GLU and TG, which indicates that dietary supplementation of this fiber exerts no adverse effects on lipid absorption in growing pigs. Meanwhile, serum CAT concentration was significantly increased in growing pigs on day 30. Although CAT does not directly participate in nitrogen metabolism, its role in scavenging H_2_O_2_, protecting nitrogen-metabolizing enzymes, and maintaining redox homeostasis may indirectly contribute to the normal progression of nitrogen metabolism [43].

### 4.4. Effects of Partial Replacement of Wheat Bran with PWCF on Nitrogen Metabolism in Growing Pigs

BUN is negatively correlated with dietary efficiency and lean tissue deposition [44]. Therefore, plasma BUN concentration serves as an indicator of dietary protein supply and utilization [45]. Supplementation of PWCF in the present study reduced serum BUN concentration, which is consistent with the findings of Malmlöf and Lenis et al., who reported that feeding high-fiber diets decreased the postprandial mean concentrations of urea in portal and arterial blood in pigs [46,47]. However, the results of the present study are also in contrast to those of Van Der Meulen (1997), who demonstrated that when corn starch was completely replaced with raw potato starch in growing pigs, the postprandial mean concentrations of urea in portal and arterial blood were increased compared with the low-fiber control group [48]. Changes in blood urea concentration depend on the dietary protein level and the fermentability of DF [49]. Therefore, based on the concept of ideal protein ratio, the use of DF combined with a reduced dietary protein level may lower the BUN concentration, thereby decreasing urea excretion via urine. In summary, compared with other DF, PWCF can effectively replace wheat bran and reduce the content of BUN in the serum of growing pigs.

The BUN/creatinine ratio is considered a sensitive indicator for evaluating protein catabolism, and its variation depends on the balance between BUN production and creatinine excretion [50]. In the present study, no significant difference in the BUN/creatinine ratio was observed between the two groups on day 30, whereas the ratio was significantly lower in the FF group on day 60. This change was primarily driven by the declining trend in BUN, while creatinine concentration remained stable throughout the experimental period. This indicates that the decline in this ratio might be attributed to the reduction in protein metabolism [51]. Mechanistically, long-term FF treatment may suppress muscle protein degradation or amino acid deamination, thereby reducing ammonia production and subsequently decreasing hepatic urea synthesis, which ultimately leads to a lower BUN concentration. However, further studies are needed to confirm this hypothesis, as nitrogen balance, urinary nitrogen excretion, and hepatic metabolism were not directly measured in the present study.

As a precursor for urea synthesis, blood ammonia can help explain the mechanism underlying the decrease in BUN [52]. In the present study, blood ammonia concentration did not differ significantly between the two groups and did not decrease in parallel with BUN. This may suggest that reduced ammonia generation was not the sole direct cause of the decline in BUN. At the same time, the stable blood ammonia concentration indicates that FF treatment did not impair hepatic ammonia detoxification capacity, thereby maintaining a safe and stable nitrogen metabolic state.

The plasma amino acid profile directly reflects amino acid metabolic homeostasis [53]. On day 30, FF treatment only tended to reduce total amino acid concentration, indicating that the animals may have maintained metabolic balance through compensatory regulation. However, on day 60, TFAA concentration was significantly reduced, with threonine showing a particularly marked decline. The decrease in total amino acids may suggest alterations in amino acid metabolism or utilization. Potential explanations could include changes in amino acid transport, intestinal absorption, or metabolic partitioning; however, these mechanisms were not directly assessed in the present study and therefore remain speculative. The concurrent reductions in total amino acids and BUN may indicate an influence of PWCF supplementation on nitrogen metabolism, although the underlying physiological mechanisms remain unclear. In addition, the decrease in essential amino acids may reflect altered amino acid availability, but its effects on protein metabolism require further investigation [54]. As an essential amino acid, threonine plays a critical role in intestinal health because it is a major constituent of mucin glycoproteins secreted by goblet cells [55]. Therefore, the reduced serum threonine concentration observed in the FF group may also reflect altered intestinal utilization associated with mucin synthesis and turnover. However, this possibility was not directly evaluated in the present study and requires further investigation.

In summary, FF treatment reduced BUN concentration and the BUN/creatinine ratio, indicating altered nitrogen metabolism. In addition, FF treatment was associated with lower serum TFAA and threonine concentrations, suggesting changes in amino acid utilization and metabolic homeostasis. Notably, blood ammonia concentration remained unchanged, indicating that FF supplementation did not adversely affect nitrogen metabolic status.

### 4.5. Effects of Partial Replacement of Wheat Bran with PWCF on Gut Microbiota in Growing Pigs

The gut microbial community is composed of various bacterial species in specific proportions, among which interspecific interactions constrain each other’s functions and enable mutual dependence to establish an ecological balance [56]. The gut microbiota plays a pivotal role in the interactions between diet and host physiology and represents one of the most important determinants of intestinal health [57]. DF acts as a substrate during fermentation and facilitates the proliferation of selective microbiota, thereby leading to alterations in the composition of the gut microbiota [24]. It has been well established that DF exerts a positive effect on maintaining the diversity of the gut microbial community and intestinal health in pigs [58]. PWCF is rich in hemicellulose dominated by xylan, which has a main chain composed of xylose residues, simple side chains, and a low degree of branching, making it an excellent carbon source for intestinal fiber-degrading bacteria [59].

Compared with the CT group, the FF group exhibited a higher relative abundance of *Treponema*, Lachnospiraceae, and Prevotellaceae. *Treponema* is a typical fiber-degrading spirochete whose genome is enriched in coding genes for xylanase, endoglucanase, and other hydrolases, enabling it to efficiently hydrolyze the xylan backbone. The high xylan content in poplar wood provides a specific substrate for this genus, allowing it to dominate the fiber-degrading ecological niche [60]. Although wheat bran also contains arabinoxylan, it has numerous and highly branched side chains, making it more readily utilized by rapidly fermenting bacteria (e.g., *Lactobacillus*, Ruminococcaceae), and thus it exhibits lower selectivity for such fiber-degrading bacteria than the high xylan in poplar wood [61]. Meanwhile, PWCF contains a certain proportion of lignin yet does not reach a high degree of lignification; it is mainly distributed in the intercellular layers and cell corners, forming a “fiber–lignin” composite structure [62]. Prevotellaceae is sensitive to fluctuations in ammonia concentration and pH [63]. Moderate lignin retards protein degradation and reduces ammonia accumulation, thus favoring its growth more, whereas wheat bran, with a high protein content and rapid fermentation, easily leads to elevated ammonia concentrations and inhibits Prevotellaceae [64].

Relevant studies have demonstrated that *Treponema*, Lachnospiraceae, and Prevotellaceae are common xylan-degrading genera and families with abundant xylanases, indicating that supplementation with PWCF can effectively increase the relative abundance of DF-degrading genera [60,65,66,67]. Wang et al. also reported similar findings that the proportions of Prevotellaceae and Lachnospiraceae are positively correlated with DF intake. These bacterial families are associated with the fermentation of plant-derived non-starch polysaccharides into short-chain fatty acids (SCFAs). *Treponema* has been reported to participate in the degradation of dietary fiber. However, fermentation metabolites, intestinal pH, pathogen abundance, nutrient absorption, and intestinal health indicators were not evaluated in the present study [68]. Therefore, whether the increased abundance of *Treponema* resulted in functional changes in the intestinal environment remains to be determined.

Likewise, several studies have demonstrated that DF supplementation in the diet promotes the growth of beneficial bacteria (e.g., Lachnospiraceae and Prevotellaceae) and inhibits the growth of pathogenic bacteria, thereby exerting a certain anti-inflammatory effect. This finding is consistent with the results of the present study: the proportion of Lachnospira in the FF group was significantly higher than that in the CT group, suggesting that DF supplementation may influence the composition of the gut microbial community. However, its effects on intestinal inflammation and microbial dysbiosis were not directly assessed in the present study [69,70].

The present study only analyzed the changes in the relative abundance of *Treponema*, Lachnospiraceae, and Prevotellaceae, but did not determine their core metabolites, especially SCFAs such as acetic acid, propionic acid, and butyric acid, making it impossible to directly verify whether the alterations in microbial abundance are actually translated into differences in metabolic functions. These results indicated that PWCF can alter the composition of the gut microbiota and increase the relative abundance of bacterial genera capable of degrading PWCF, whereas whether it exerts beneficial effects on intestinal barrier function requires further investigation.

## 5. Conclusions

In conclusion, replacing 2% wheat bran with PWCF in diets for growing pigs had no adverse effects on growth performance, nutrient digestibility, or health-related indicators, while influencing serum nitrogen-related parameters and gut microbiota composition. Further studies evaluating different inclusion levels are required to determine the dose-dependent effects and practical application potential of PWCF.

## Figures and Tables

**Table 1:** Ingredient composition and calculated nutrient levels of the basal diet (as-fed basis, %).

Items	Treatment Groups	
	CT	FF
Corn	63.87	63.87
Soybean meal, 46% CP	19.00	19.00
Rice bran meal	8.00	8.00
Wheat bran	4.00	2.00
Soybean oil	1.90	1.90
Dicalcium phosphate	0.18	0.18
Limestone	1.50	1.50
L-Lysine	0.70	0.70
DL-Methionine	0.05	0.05
L-Threonine	0.14	0.14
L-Tryptophan	0.01	0.01
Salt	0.40	0.40
Phytase	0.02	0.02
Vitamin premix ^1^	0.03	0.03
Mineral premix ^1^	0.20	0.20
Poplar wood composite fiber	0.00	2.00
Total	100.00	100.00
Nutrient composition		
Crude protein ^2^	15.45	15.41
Net energy, kcal/kg ^3^	2475	2475
Calcium ^2^	0.67	0.68
Total phosphorus ^2^	0.51	0.51
Neutral detergent fiber ^3^	12.24	13.25
SID Lysine ^3^	1.00	1.00
SID Methionine ^3^	0.28	0.28
SID Threonine ^3^	0.60	0.60
SID Tryptophan ^3^	0.16	0.16

SID = Standardized ileal digestibility. ^1^ Premix supplied per kilogram of diets: vitamin A, 7245 IU; vitamin D_3_, 1470 IU; vitamin E, 40 IU; vitamin K_3_, 1.79 mg; vitamin B_1_, 2.14 mg; vitamin B_2_, 4.37 mg; vitamin B_6_, 2.68 mg; vitamin B_12_, 0.024 mg; niacin, 25 mg; calcium pantothenate, 15 mg; folic acid, 4.15 mg; 2%biotin, 0.42 mg; iron (FeSO_4_·H_2_O), 150 mg; manganese (MnSO_4_·H_2_O), 100 mg; copper (CuSO_4_·5H_2_O), 23 mg; selenium (Na_2_SeO_3_), 0.5 mg; cobalt (CoCl_2_), 1 mg; iodine (Ca(IO_3_)_2_), 23 mg; Zinc 79 mg. ^2^ Values were analyzed. ^3^ Values were calculated.

**Table 2:** Nutrient composition and fiber physical characteristics of the poplar wood composite fiber (PWCF) (air dry basis, %).

Items	Composition
Nutrient composition	
Moisture	5.42
Crude protein	3.55
Crude fiber	44.25
Neutral detergent fiber (NDF)	70.51
Acid detergent fiber (ADF)	59.55
Ether extract (EE)	2.65
Nitrogen-free extract	14.00
Ash	30.13
Calcium	1.50
Phosphorus	0.03
Fiber physical characteristics	
Water-holding capacity (g/mL)	6.25
Swelling capacity (g/mL)	6.17

**Table 3:** Growth performance of growing pigs fed diets in which wheat bran was partially replaced by poplar wood composite fiber.

Items	Treatment Groups		SEM	*p*-Value
	CT	FF		
Body weight, kg				
0 d	47.4	47.7	0.81	0.511
30 d	71.9	71.5	1.18	0.777
60 d	99.3	99.2	1.85	0.965
Average daily gain, g/d				
1~30 d	817	791	29.3	0.584
31~60 d	911	923	31.6	0.746
1~60 d	864	861	27.7	0.875
Average daily feed intake, g/d				
1~30 d	2151	2104	62.9	0.632
31~60 d	2656	2695	65.3	0.819
1~60 d	2403	2400	58.7	0.969
Average daily feed intake: Average daily gain (F: G)				
1~30 d	2.63	2.67	0.05	0.586
31~60 d	2.92	2.93	0.05	0.870
1~60 d	2.78	2.80	0.04	0.667

CT = basal diet; FF = CON + 2% poplar wood composite fiber (replacing 2% wheat bran). *n* = 5.

**Table 4:** Effects of partial replacement of wheat bran with poplar wood composite fiber on apparent total tract digestibility in growing pigs.

Items	Treatment Groups		SEM	*p*-Value
	CT	FF		
Dry matter, %	79.2	78.8	1.16	0.825
Crude protein, %	75.3	77.2	1.71	0.464
Calcium, %	34.8	36.4	4.49	0.810
Phosphorus, %	35.8	39.0	4.06	0.590
Ether extract, %	72.9	75.0	2.20	0.512
Gross energy, %	80.2	80.6	1.18	0.807

CT = basal diet; FF = CON + 2% poplar wood composite fiber (replacing 2% wheat bran). *n* = 5.

**Table 5:** Effects of partial replacement of wheat bran with poplar wood composite fiber on serum biochemical parameters in growing pigs.

Items	Treatment Groups		SEM	*p*-Value
	CT	FF		
Glucose, mmol/L				
30 d	5.47	4.97	0.272	0.210
60 d	4.44	4.56	0.187	0.656
Triglyceride, mmol/L				
30 d	0.22	0.27	0.030	0.115
60 d	0.22	0.29	0.025	0.194
Total protein, mg/mL				
30 d	53.2	52.2	1.97	0.715
60 d	56.6	58.5	1.84	0.498
Blood urea nitrogen, mmol/L				
30 d	0.57	0.68	0.053	0.197
60 d	0.72	0.58	0.057	0.084
Creatinine, μmol/mL				
30 d	0.22	0.22	0.009	0.576
60 d	0.23	0.21	0.012	0.490
Blood ammonia, μmol/mL				
30 d	0.33	0.31	0.013	0.233
60 d	0.34	0.30	0.024	0.194
Blood urea nitrogen/Creatinine				
30 d	2.71	3.10	0.310	0.388
60 d	3.22	2.64	0.190	0.048

CT = basal diet; FF = CON + 2% poplar wood composite fiber (replacing 2% wheat bran). *n* = 5.

**Table 6:** Effects of partial replacement of wheat bran with poplar wood composite fiber on serum-free amino acids in growing pigs.

Items	Treatment Groups		SEM	*p*-Value
	CT	FF		
30 d (mg/L)				
Aspartic acid	8.49	8.04	0.692	0.660
Glutamic acid	40.23	37.24	2.314	0.383
Serine	24.75	22.01	1.778	0.324
Histidine	54.08	52.08	2.305	0.562
Glycine	94.50	80.56	6.504	0.160
Threonine	25.59	27.14	3.771	0.779
Arginine	43.34	36.45	3.819	0.219
Alanine	61.89	54.66	3.532	0.165
Tyrosine	34.41	29.49	2.793	0.234
Valine	41.64	40.93	1.660	0.776
Methionine	17.95	18.42	1.582	0.834
Isoleucine	21.58	21.72	1.226	0.937
Phenylalanine	25.41	30.44	1.924	0.082
Lysine	40.93	39.51	2.705	0.715
Leucine	32.09	30.37	1.761	0.505
Total free amino acid	572.35	526.09	13.221	0.059
60 d (mg/L)				
Aspartic acid	8.32	6.43	0.772	0.102
Glutamic acid	34.42	26.46	2.968	0.074
Serine	17.40	15.39	1.470	0.361
Histidine	12.22	10.50	1.308	0.383
Glycine	78.54	68.74	4.615	0.154
Threonine	47.64	20.59	4.432	<0.001
Arginine	36.38	31.77	5.268	0.545
Alanine	39.68	30.33	3.252	0.065
Tyrosine	21.90	20.67	2.742	0.757
Valine	34.56	32.68	1.889	0.497
Methionine	9.33	11.86	1.513	0.252
Isoleucine	15.33	14.47	1.359	0.692
Phenylalanine	11.64	11.62	1.123	0.991
Lysine	36.30	29.31	4.487	0.329
Leucine	28.82	25.33	1.852	0.228
Total free amino acid	433.97	357.65	15.190	0.010

CT = basal diet; FF = CON + 2% poplar wood composite fiber (replacing 2% wheat bran). *n* = 5.

**Table 7:** Effects of partial replacement of wheat bran with poplar wood composite fiber on immune parameters in growing pigs.

Items	Treatment Groups		SEM	*p*-Value
	CT	FF		
Immunoglobulin G, mg/mL				
30 d	34.9	35.0	0.546	0.853
60 d	34.4	34.0	0.864	0.644
Immunoglobulin A, μg/mL				
30 d	181	182	3.99	0.818
60 d	184	182	2.92	0.571
Immunoglobulin M, mg/mL				
30 d	3.67	3.77	0.071	0.356
60 d	3.60	3.45	0.088	0.282

CT = basal diet; FF = CON + 2% poplar wood composite fiber (replacing 2% wheat bran). *n* = 5.

**Table 8:** Effects of partial replacement of wheat bran with poplar wood composite fiber on serum biochemical parameters in growing pigs.

Items	Treatment Groups		SEM	*p*-Value
	CT	FF		
Malondialdehyde, nmol/mL				
30 d	4.18	3.89	0.157	0.120
60 d	3.84	3.73	0.148	0.642
Catalase, U/mL				
30 d	9.80	10.87	0.224	0.013
60 d	10.34	10.61	0.420	0.615
Superoxide dismutase, U/mL				
30 d	9.31	10.82	0.803	0.230
60 d	8.70	9.12	0.645	0.656

CT = basal diet; FF = CON + 2% poplar wood composite fiber (replacing 2% wheat bran). *n* = 5.

## Data Availability

The 16S rRNA raw data supporting the findings of this study are available in the NCBI database at https://www.ncbi.nlm.nih.gov/bioproject/PRJNA1468394/ (accessed on 20 May 2026), accession number PRJNA1468394. The data are publicly accessible and can be requested from the corresponding author for any additional information or clarification.

## Abbreviations

The following abbreviations are used in this manuscript:

ADF	Acid detergent fiber
ADFI	Average daily feed intake
ADG	Average daily gain
AIA	Acid-insoluble ash
AOAC	Association of Official Analytical Chemists
ASV	Amplicon sequence variants
ATTD	Apparent total tract digestibility
BUN	Blood urea nitrogen
BW	Body weight
CAT	Catalase
CF	Crude fiber
CP	Crude protein
CT	Control group
DM	Dry matter
FF	Fiber treatment group
F: G	Feed-to-gain ratio
GE	Gross energy
GLU	Glucose
IgA	Immunoglobulin A
IgG	Immunoglobulin G
IgM	Immunoglobulin M
MDA	Malondialdehyde
NDF	Neutral detergent fiber
PCoA	Principal coordinate analysis
PWCF	Poplar wood composite fiber
SCFAs	Short-chain fatty acids
SOD	Superoxide dismutase
TFAA	Total free amino acid
TG	Triglyceride
TP	Total protein
